# Effect of Spray Characteristic Parameters on Friction Coefficient of Ultra-High-Strength Steel against Cemented Carbide

**DOI:** 10.3390/ma17194867

**Published:** 2024-10-03

**Authors:** Bangfu Wu, Minxiu Zhang, Biao Zhao, Benkai Li, Wenfeng Ding

**Affiliations:** 1 National Key Laboratory of Science and Technology on Helicopter Transmission, College of Mechanical and Electrical Engineering, Nanjing University of Aeronautics and Astronautics, Nanjing 210016, China; wubangfu@nuaa.edu.cn (B.W.); zhangmx2000@nuaa.edu.cn (M.Z.); zhaobiao@nuaa.edu.cn (B.Z.); 2 School of Mechanical and Automotive Engineering, Qingdao University of Technology, Qingdao 266520, China

**Keywords:** ultra-high-strength steel, spray characteristic parameters, cooling condition, liquid film, friction coefficient

## Abstract

Ultra-high-strength steels have been considered an essential material for aviation components owing to their excellent mechanical properties and superior fatigue resistance. When machining these steels, severe tool wear frequently results in poor surface quality and low machining efficiency, which is intimately linked to the friction behavior at the tool–workpiece interface. To enhance the service life of tools, the adoption of efficient cooling methods is paramount. However, the understanding of friction behavior at the tool–workpiece interface under varying cooling conditions remains limited. In this work, both air atomization of cutting fluid (AACF) and ultrasonic atomization of cutting fluid (UACF) were employed, and their spray characteristic parameters, including droplet size distribution, droplet number density, and droplet velocity, were evaluated under different air pressures. Discontinuous sliding tests were conducted on the ultra-high-strength steel against cemented carbide and the effect of spray characteristic parameters on the adhesion friction coefficient was studied. The results reveal that ultrasonic atomization significantly improved the uniformity of droplet size distribution. An increase in air pressure resulted in an increase in both droplet number density and droplet velocity under both AACF and UACF conditions. Furthermore, the thickness of the liquid film was strongly dependent on the spray characteristic parameters. Additionally, UACF exhibited a reduction of 4.7% to 9.8% in adhesion friction coefficient compared to AACF. UACF provided the appropriate combination of spray characteristic parameters, causing an increased thickness of the liquid film, which subsequently exerted a positive impact on reducing the adhesion friction coefficient.

## 1. Introduction

Ultra-high-strength steels, renowned for their high strength, high toughness, and superior fatigue resistance, have been widely utilized in aviation transmission gears and aircraft landing gear [1,2,3]. Currently, mechanical cutting is the primary method for machining ultra-high-strength steel parts. However, the excellent physical and mechanical properties of these steels lead to the rapid wear of tools, significantly affecting the surface quality, machining efficiency, and costs [4,5]. Tool wear is primarily influenced by the friction and wear behaviors in the cutting zone [6,7,8]. Therefore, it is meaningful to investigate the tribological behavior between workpiece material and tool material under different cooling conditions, with the aim of inhibiting rapid tool wear.

The reciprocating ball–disc sliding tests have been chosen by many researchers for evaluating the tribological behavior between the workpiece and the tool [9,10]. Li et al. [11] investigated the tribological behavior of 7050 aluminum alloy against YG8 cemented carbide under different sliding velocities and loads. They found that the friction coefficient exhibited a decreasing trend as the sliding velocity and load increased. Li et al. [12] carried out the reciprocating sliding tests using TiAlN-coated and AlTiN-coated cutting tools and found that the TiAlN coating exhibited a better wear resistance than the AlTiN coating. Jamil et al. [13] studied the effect of different cooling media (e.g., ethanol, ester oil, and dry ice) on the tribological behavior between Ti-6Al-4V alloy and WC cemented carbide. They demonstrated that the mixtures of ethanol, ester oil, and dry ice could achieve a lower friction coefficient and wear rate compared to single cooling media. García-Martínez et al. [14] compared the tribological behavior of copper–nickel alloy against a coated tool under three cooling conditions including dry, flood, and low initial lubrication. The results indicated that the low initial lubrication condition could reduce the friction coefficient due to the existence of a lubrication layer formed by the mixture of oil and debris. El-Tayeb et al. [15] investigated the wear behavior of Ti54 alloy against the cemented carbide under cryogenic conditions and reported that abrasion and delamination were the predominant wear modes. However, it was noted that the reciprocating sliding test can’t reflect the real contact conditions of the workpiece tool during the cutting process due to the repeated contact between the ball surface and the workpiece surface.

To solve this problem, scholars have developed the open ball–disc tribo-system to achieve the single sliding of a ball on the fresh surface of the workpiece [16,17,18,19]. Bonnet et al. [20] developed a new friction model to calculate the friction coefficient between 316L stainless steel and TiN-coated carbide during the dry sliding process. They concluded that the friction coefficient was strongly affected by the sliding velocity. Klinkova et al. [21] carried out the sliding tests of carbon fiber reinforced plastics (CFRP) with cemented carbide under dry conditions and revealed that the friction coefficient experienced a remarkable reduction from 0.25 to 0.1 as the sliding velocity varied from 10 m/min to 120 m/min. On the contrary, Mondelin et al. [22] insisted that the friction coefficient remained largely unaffected by variations in both sliding velocity and contact pressure during the sliding process of the CFRP with monocrystalline diamond. Abdelali et al. [23] pointed out that varying sliding velocities led to distinct tribological behaviors, which can be distinguished by examining the friction coefficient, heat partition coefficient, and material adhesion. Xu et al. [24,25] performed friction experiments on CFRP material and concluded that the elastic recovery of CFRP material affected the friction coefficient and the generation of friction heat.

Many efforts have been undertaken to eliminate the adverse effect of severe friction during the sliding process. Claudin et al. [26] conducted an investigation into the tribological behavior of AISI4140 steel sliding against TiN-coated carbide under dry and straight oil conditions. Their finding indicated that the straight oil can effectively penetrate the contact interface, reducing the friction coefficient. Fersi et al. [27] conducted a friction test of Ti-6Al-4V alloy with WC cemented carbide under various cooling conditions, including dry, emulsion, and cryogenic conditions. They discovered that the application of cryogenic conditions led to a notable reduction in the friction coefficient compared to the dry and emulsion conditions. The authors further explained that superior cooling ability under cryogenic conditions lowered the temperature, subsequently reducing the material adhesion of the Ti-6Al-4V alloy, resulting in a low friction coefficient. However, different results were reported by Courbon et al. [28], who investigated the influence of cryogenic conditions on the friction behavior of Ti-6Al-4V alloy against WC cemented carbide. They demonstrated that the cryogenic condition did not affect the friction coefficient and material adhesion. Etri et al. [29] carried out tribological tests on Ti-6Al-4V alloy against WC cemented carbide using minimal quantity lubrication (MQL) with different nanoparticles (i.e., graphene and hBN). Their research indicated that hybrid nanofluid (graphene + hBN + vegetable oil) attained a low friction coefficient and wear rate due to its superior heat conductivity and lubrication capability. Demirsoz et al. [30] studied the effect of different cooling conditions (i.e., dry, MQL, cryogenic, and cryo-MQL) on the tribological performance of 316L stainless steel against 100 Cr6 alloy. They concluded that the synergistic effect of cooling and lubrication in cryo-MQL significantly contributed to lowering the friction coefficient and enhancing the wear resistance of 316L stainless steel. Furthermore, Behera et al. [31] and Chetan et al. [32] applied MQL to the friction tests and found that the friction coefficient was strongly influenced by the air pressure and flow rate.

In summary, previous studies have mainly focused on the continuous contact situation, which was suitable for describing the friction behavior during the continuous cutting processes, such as turning. However, for the intermittent cutting behavior in a milling operation, the continuous contact condition was not applicable. Therefore, the tribological behavior under the discontinuous contact condition needs to be further studied. In addition, the implementation of effective cooling and lubrication conditions significantly contributed to the reduction of the friction coefficient at the contact interface and the enhancement of the lubrication state. Especially, during the milling process of ultra-high-strength steel, atomization modes of cutting fluid have exhibited excellent cooling and lubrication performance [33]. The liquid film generated by droplets is the main factor determining the lubrication state, and the film’s characteristics are primarily affected by the spray characteristic parameters. Therefore, the novelty of this work lies in studying the effect of spray characteristic parameters on the tribological behavior of ultra-high-strength steel against cemented carbide during the discontinuous sliding process.

In this study, air atomization of cutting fluid (AACF) and ultrasonic atomization of cutting fluid (UACF) were applied to obtain the different spray characteristic parameters. The effect of different spray characteristic parameters on the liquid film thickness was investigated. Subsequently, the relationship between liquid film thickness and adhesion friction coefficient was discussed. The remainder of this paper is organized as follows. Section 2 presents the cooling system and the characterization method for spray characteristic parameters (i.e., droplet size distribution, droplet velocity, and droplet number density). Section 3 introduces the experimental setup and materials used for the discontinuous sliding test. Additionally, a calculation model of the adhesion friction coefficient during the discontinuous sliding process was developed. Section 4 analyzes the influence of air pressure on the spray characteristic parameters, the thickness of the liquid film, and the adhesion friction coefficient.

## 2. Cooling Methods

### 2.1. Cooling System

**Figure 1:**
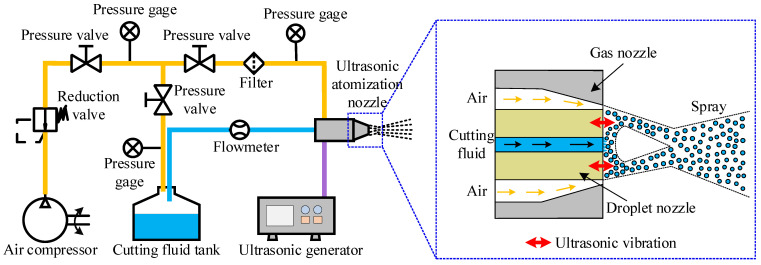
Schematic diagram of the cooling system for AACF and UACF conditions.

A self-developed cooling system was utilized to implement these two cooling conditions, namely AACF and UACF. This system consisted of an ultrasonic atomization nozzle, an air compressor, a cutting fluid tank, and an ultrasonic generator, as depicted in Figure 1. Further details regarding the cooling system can be found in the previous study [33]. The air compressor can provide high-pressure air for the ultrasonic atomization nozzle. Additionally, a small amount of compressed air forced the cutting fluid from the cutting fluid tank to the inside of the nozzle. The ultrasonic atomization nozzle played two crucial roles. Firstly, it dispersed the cutting fluid into fine droplets through the capillary wave effect [34]. Secondly, these droplets were subsequently mixed with a high-velocity airflow at the nozzle outlet, forming a uniform spray. The ultrasonic atomization process operated at a frequency of 50.5 kHz. The AACF condition could be achieved by stopping the ultrasonic generator. In this case, the cutting fluid was dispersed into small droplets by the shearing effect of airflow [35]. It was noted that different atomization methods affected the spray characteristic parameters, resulting in different liquid film characteristics.

### 2.2. Characterization of Spray Characteristic Parameters

In the machining process, the droplet forms a liquid film, which serves to enhance both cooling and lubrication capabilities. The spray characteristic parameters, including droplet size distribution, droplet velocity, and droplet number density, are crucial factors influencing the formation of the liquid film. Consequently, the impact of air pressure on these parameters was investigated. The droplet deposition method was employed to obtain the droplet size distribution and density number density. The droplet number density is defined as the number of droplets per unit area. Subsequently, the droplet velocity was calculated based on the force balance equation.

**Figure 2:**
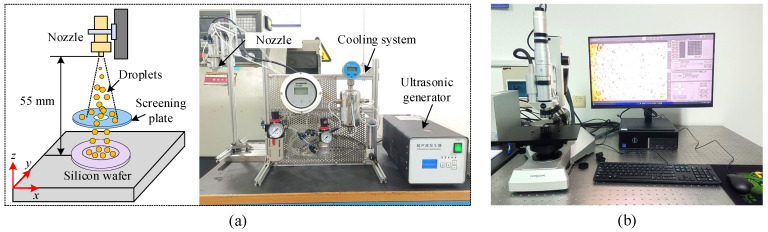
Experimental setup for droplet measurement: (**a**) droplet deposition device and (**b**) optical microscope.

**Figure 3:**
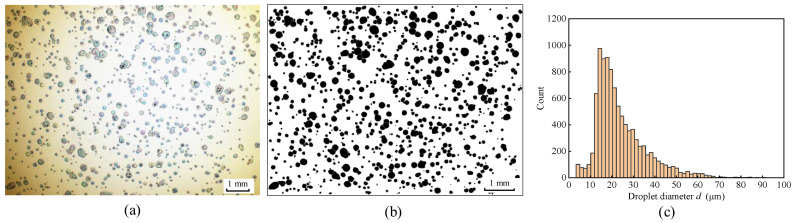
The recognition process of droplet image: (**a**) original image, (**b**) binary image, and (**c**) statistics results of droplet size distribution.

Figure 2 illustrates the setup for droplet measurement. A polished silicon wafer was utilized to collect the droplets. The spray distance between the nozzle outlet and the silicon wafer was maintained at 55 mm. Moreover, a screening plate featuring a hole was positioned between the nozzle outlet and the silicon wafer to avoid droplet overlap (Figure 2a). The droplets discharged from the nozzle outlet passed through the screening plate and were then deposited on the silicon wafer. Subsequently, high-resolution droplet images were captured using an optical microscope (Figure 2b). The original droplet image was identified using Image J fiji software, as shown in Figure 3. The recognition procedure included image binarization, droplet boundary detection and filling, image filtering, image scale conversion, and statistical analysis of the droplet sizes. Based on the statistical results gathered from 10 distinct droplet images, the droplet size distribution and droplet number density were derived.

During the formation of the spray under AACF and UACF, the airflow provided the driving force for the droplet movement. To obtain the droplet velocity, the force situation of an individual droplet within the airflow was analyzed. The force balance equation governing the droplet dynamics can be formulated as follows [36]:

(1) $$\rho_{d}V_{d}\frac{dv_{d}}{dt}=V_{d}(\rho_{d}-\rho_{g})g-\frac{1}{2}\rho_{g}A_{s}C_{d}(v_{d}-v_{g})\left|v_{d}-v_{g}\right|$$

where *ρ*_d_ and *ρ*_g_ denote the densities of the droplet and air, respectively. *v*_d_ and *v*_g_ represent the velocities of the droplet and air, respectively. *V*_d_ is the volume of the droplet, *g* stands for the gravitational acceleration, and *A*_s_ is the cross-sectional area of the droplet. The drag coefficient *C*_d_ for a spherical droplet can be estimated by Equation (2) [37]:

(2) $$C_{d}=0.28+\frac{6\sqrt{Re_{r}}+21}{Re_{r}}$$

where *Re*_r_ represents the relative Reynolds number, which is defined as follows:

(3) $$Re_{r}=\frac{\rho_{g}d\left|v_{g}-v_{d}\right|}{\mu_{g}}$$

where *μ*_g_ signifies the dynamic viscosity of the air.

Table 1 lists the detailed cooling parameters for the AACF and UACF processes. A 12% concentration of cutting fluid was obtained by mixing a water-soluble synthetic (Castrol 9954) with water. The measured cutting fluid density is 995 kg/m^3^. The air has a density of 1.29 kg/m^3^ and a dynamic viscosity of 1.82 × 10^−5^ Pa·s, respectively. The effect of different air pressures on air velocity has been studied in previous work [33]. Based on the aforementioned parameters, the droplet velocities under different air pressures were determined by solving Equation (1).

## 3. Experimental Setup and Method

### 3.1. Discontinuous Sliding Test

**Figure 4:**
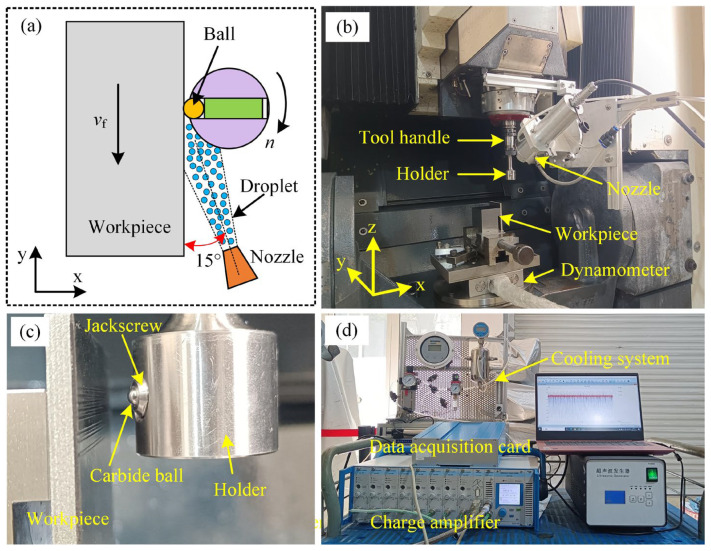
Experimental setup for the discontinuous sliding test under different cooling conditions: (**a**) schematic diagram of the discontinuous sliding process, (**b**) open ball–disc tribometer system, (**c**) holder, and (**d**) force measurement system and cooling system.

The discontinuous sliding tests under various cooling conditions were carried out on a precision machining center (DMG) equipped with a developed open ball–disc tribometer system (Figure 4). Figure 4b depicts the specific configuration of the open ball–disc tribometer system, comprising a ball, a holder, a tool handle, a workpiece, and a piezoelectric dynamometer. The ball was securely fastened to the side of the holder via a jackscrew and bolt (Figure 4c). This holder was then mounted onto the tool handle, which has the capability to rotate synchronously with the spindle of the machine tool. During the test, the ball made a single contact with the workpiece as the spindle completed a full rotation cycle. Furthermore, the feed motion of the workpiece ensured that the ball only slid on a fresh surface of the workpiece. The workpiece was placed on the piezoelectric dynamometer, and the force acting upon it was measured by the force measurement system (Kistler, Winterthur, Switzerland), featuring a three-component dynamometer, charge amplifier, and data acquisition card, as shown in Figure 4d. Additionally, the ultrasonic atomization nozzle was mounted on the machine spindle to provide different cooling conditions for the contact interface between the ball and the workpiece.

**Figure 5:**
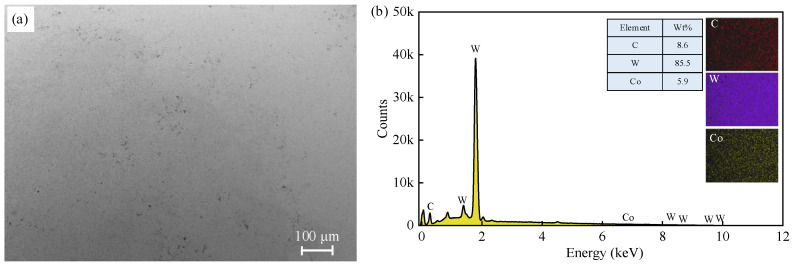
WC cemented carbide ball: (**a**) SEM image of microstructure and (**b**) EDS mapping.

**Figure 6:**
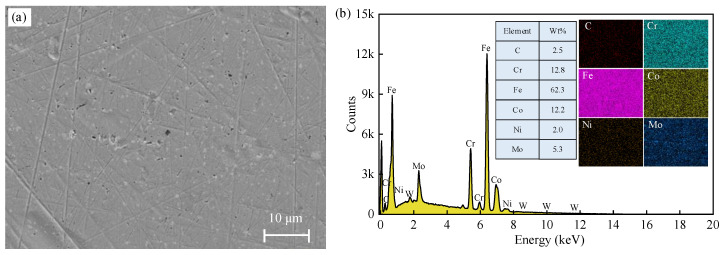
Ultra-high-strength steel: (**a**) SEM image of microstructure and (**b**) EDS mapping.

The ball employed in this work was a commercial WC-Co cemented carbide ball with a diameter of 3.17 mm. Figure 5 displays the microstructure and chemical composition of the cemented carbide ball. The measured surface roughness *S*_a_ was 0.45 ± 0.02 μm and the hardness was 76 ± 2 HRC. The tested workpiece was 15Cr14Co12Mo5Ni2 ultra-high-strength steel with dimensions of 60 mm × 60 mm × 5 mm. The workpiece surface was polished to eliminate the effect of surface roughness on the sliding process. The surface roughness *S*_a_ of the polished surface has reached 0.17 ± 0.04 μm. Figure 6 presents the microstructure of the polished surface and the chemical composition of the workpiece. Table 2 lists the mechanical properties of 15Cr14Co12Mo5Ni2 ultra-high-strength steel [38].

The sliding test was performed with a sliding velocity of 50 m/min and a feed rate of 0.01 mm/z. Additionally, to ensure that the cemented carbide ball experienced a constant load from the workpiece, the initial indentation depth was maintained at 0.1 mm. The spray distance between the ball surface and the nozzle outlet was fixed at 55 mm. The angle between the nozzle and the workpiece was 15°. Each sliding test lasted for a duration of 10 min. Three repeated experiments for each cooling parameter were conducted. After the sliding experiment, the pollutants on the workpiece surface were removed using ultrasonic cleaning with an alcohol solution. A 3D optical profilometer (Sensofar, Barcelona, Spain) was employed to detect the 3D surface morphology of the wear mark on the workpiece. Additionally, the worn surfaces of the balls were detected using the scanning electron microscope (Zeiss sigma300, Carl Zeiss AG, Oberkochen, Germany).

### 3.2. Analysis of Adhesion Friction Coefficient

The friction coefficient is commonly used to evaluate the friction behavior of frictional pairs. In the sliding process, the contact zone of frictional pairs is subjected to the tangential force and normal force. It is noted that the high contact pressure results in the plastic deformation of the workpiece material. In this case, the measured macroscopic tangential force can be comprised of an adhesion component and a ploughing component [39,40]. Therefore, the tangential force can be expressed as follows:

(4) $$F_{t}=F_{a}+F_{p}$$

where *F*_a_ is the adhesion force between the ball and the workpiece, and *F*_p_ is the ploughing force caused by the plastic deformation of the workpiece material.

Similarly, the apparent friction coefficient can be written as follows:

(5) $$\mu =\frac{F_{a}}{F_{n}}+\frac{F_{p}}{F_{n}}=\mu_{a}+\mu_{p}$$

where *F*_n_ is the normal force, *μ*_a_ is the adhesion friction coefficient, and *μ*_p_ is the ploughing friction coefficient.

Based on the above analysis, the adhesion friction coefficient *μ*_a_ can represent the real friction coefficient of the contact surface between frictional pairs. The adhesion friction coefficient can be calculated by Equation (6) [26].

(6) $$\mu_{a}=\frac{S_{1}\mu -S_{2}}{S_{2}\mu +S_{1}}$$

where *S*_1_ and *S*_2_ are the projection areas of the contact surfaces along the tangential and normal directions, respectively.

It should be noted that the plastic deformation of the workpiece material resulted in the formation of the pile-up, directly affecting the projection area of the contact surface. Specifically, the pile-up of material resulting from the previous sliding process can increase the area of the contact surface in the subsequent sliding process. Therefore, the influence of material pile-up on the adhesion friction coefficient needs to be considered during the discontinuous sliding process.

**Figure 7:**
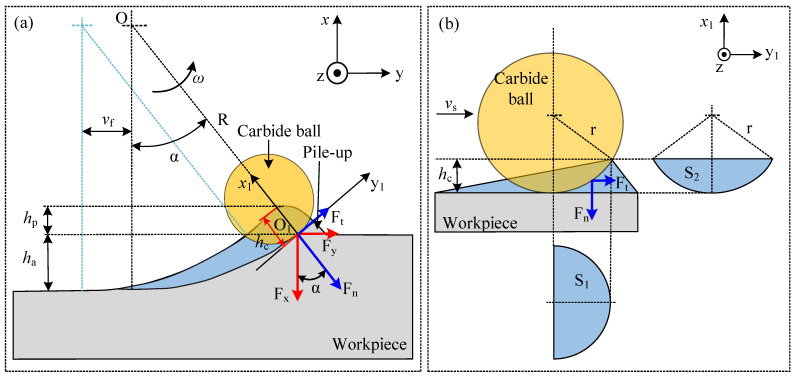
Discontinuous sliding process: (**a**) force transformation relationship under maximum sliding depth and (**b**) projection area of the contact surface in a local coordinate system.

Since the discontinuous sliding process is similar to the milling process, the sliding depth of the ball varies with the rotation angle of the holder, as shown in Figure 7. The situation that the contact surface at the maximum sliding depth was analyzed in this study. The maximum sliding depth *h*_c_ can be approximately written as [41]:

(7) $$h_{c}=v_{f}\sin\alpha$$

where *v*_f_ stands for the feed rate of the workpiece, and *α* denotes the rotation angle of the holder when the sliding depth varies from zero to its maximum value.

Based on the force transformation relationships depicted in Figure 7a, the tangential force and normal force acting on the contact surface at the maximum sliding depth can be given as follows:

(8) $$F_{t}=F_{y}\cos\alpha -F_{x}\sin\alpha$$

(9) $$F_{n}=F_{y}\sin\alpha +F_{x}\cos\alpha$$

where *F*_x_ and *F*_y_ represent the forces measured by the dynamometer in the *x* and *y* directions, respectively.

Additionally, the rotation angle of the ball under maximum sliding depth can be calculated as follows:

(10) $$\alpha =\arctan\frac{\sqrt{R^{2}-{\left(R-h_{a}-h_{p}\right)}^{2}}-v_{f}}{R-h_{a}-h_{p}}$$

where *R* represents the rotation radius of the cemented carbide ball, *h*_a_ denotes the depth of wear mark, and *h*_p_ signifies the height of pile-up of the material.

Since the rotation angle of the ball is small, the maximum sliding depth *h*_c_ can also be written as follows:

(11) $$h_{c}=v_{f}\sin\alpha =v_{f}\tan\alpha =\frac{v_{f}\sqrt{R^{2}-{\left(R-h_{a}-h_{p}\right)}^{2}}-v_{f}^{2}}{R-h_{a}-h_{p}}$$

Moreover, the projection areas of contact surfaces *S*_1_ and *S*_2_ in Figure 7b can be calculated as follows:

(12) $$S_{1}=\frac{\pi }{2}(r^{2}-{\left(r-h_{c}\right)}^{2})$$

(13) $$S_{2}=r^{2}\arccos\frac{r-h_{c}}{r}-(r-h_{c})\sqrt{r^{2}-(r-h_{c}{)}^{2}}$$

where *r* is the radius of the cemented carbide ball.

Substituting Equations (8), (9), (12) and (13) into Equation (6), the adhesion friction coefficient in the discontinuous sliding process can be expressed as follows:

(14) $$\mu_{a}=\frac{\frac{\pi }{2}(r^{2}-{\left(r-h_{c}\right)}^{2})(\frac{F_{y}\cos\alpha -F_{x}\sin\alpha }{F_{y}\sin\alpha +F_{x}\cos\alpha })-(r^{2}\arccos\frac{r-h_{c}}{r}-(r-h_{c})\sqrt{r^{2}-(r-h_{c}{)}^{2}})}{\left(r^{2}\arccos\frac{r-h_{c}}{r}-(r-h_{c})\sqrt{r^{2}-(r-h_{c}{)}^{2}})(\frac{F_{y}\cos\alpha -F_{x}\sin\alpha }{F_{y}\sin\alpha +F_{x}\cos\alpha })+\frac{\pi }{2}(r^{2}-{\left(r-h_{c}\right)}^{2}\right)}$$

**Figure 8:**
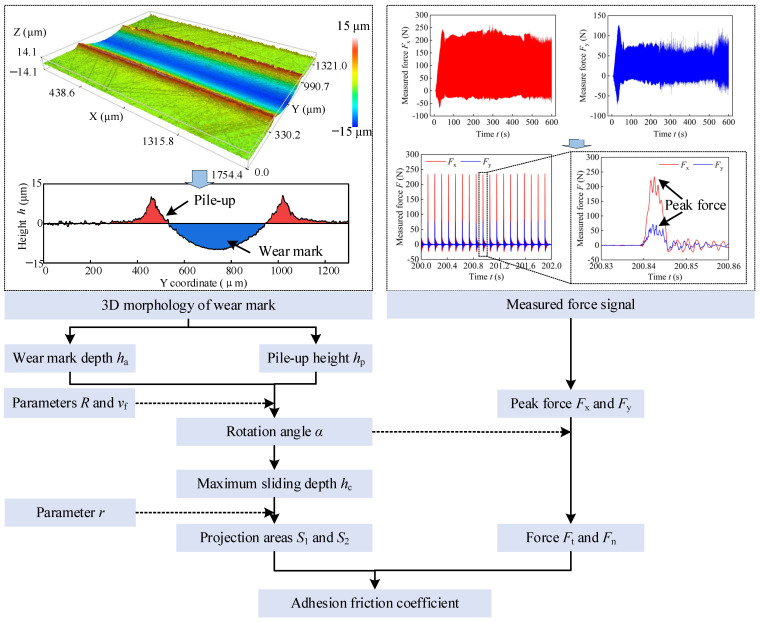
Calculation process of adhesion friction coefficient.

Equation (14) demonstrates that the adhesion friction coefficient relies on specific parameters, including the depth of the wear mark *h*_a_, the height of material pile-up *h*_p_, and the measured forces *F*_x_ and *F*_y_. These parameters can be acquired from the sliding experiment. Figure 8 illustrates the calculation process of the adhesion friction coefficient. Initially, the wear mark depth h_a_ and pile-up height h_p_ were measured through the cross-section of the wear mark. Then, the parameters of rotation angle *α*, maximum sliding depth *hc*, and projection areas *S*_1_ and *S*_2_ were calculated sequentially. To simplify the processing of measured force signal data and reduce the computational complexity, the force signal was extracted every second. Furthermore, the peak force was determined, and the tangential force *F*_t_ and normal force *F*_n_ were subsequently calculated. Finally, the adhesion friction coefficient curve was obtained, and the average adhesion friction coefficient in the steady stage was analyzed.

## 4. Results and Discussion

### 4.1. Effect of Air Pressure on Spray Characteristic Parameters

**Figure 9:**
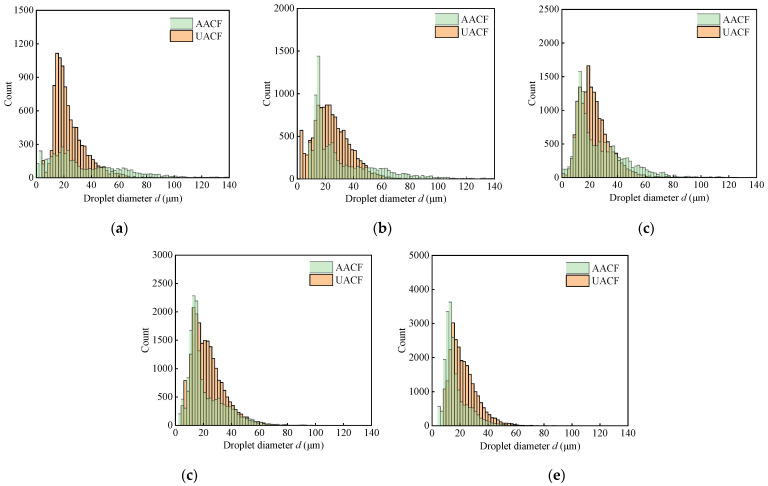
Histogram of droplet size distribution under different air pressures: (**a**) *P* = 140 kPa, (**b**) *P* = 180 kPa, (**c**) *P* = 220 kPa, (**d**) *P* = 260 kPa, and (**e**) *P* = 300 kPa.

**Figure 10:**
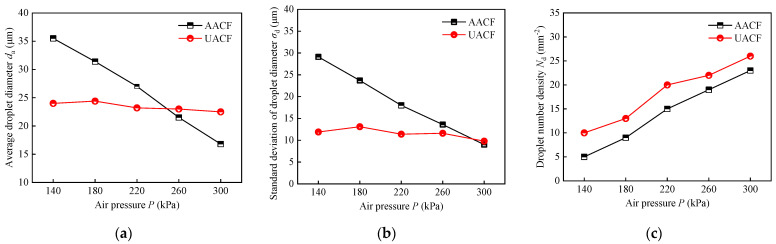
Statistical results of droplet size distribution and number density under different air pressures: (**a**) average droplet diameter *d*_a_, (**b**) standard deviation of droplet diameter *σ*_d_, and (**c**) droplet number density *N*_d_.

The histogram plots in Figure 9 clearly present the droplet size distribution at different air pressures for AACF and UACF. These histograms illustrate the correlation between the droplet count and the droplet diameter. Notably, it was observed that the majority of droplet diameters lie within the range of 0 μm to 60 μm. To quantitatively assess this droplet size distribution, both the average value and the standard deviation of the droplet diameters were employed. Furthermore, Figure 10 comprehensively depicts the effect of different air pressures on the three key parameters: the average droplet diameter, standard deviation of droplet diameter, and droplet number density.

As shown in Figure 10a, under AACF conditions, the average droplet diameter decreased linearly from 35.5 μm to 16.8 μm as the air pressure varied from 140 kPa to 300 kPa. This was because the enhanced air velocity in higher air pressure exerted a stronger shear force on the liquid surface, thereby facilitating the breakup of the liquid into smaller droplets [42]. In contrast, despite the increase in air pressure, the average droplet diameter remained virtually unchanged under UACF conditions. This was attributed to the fact that the droplet diameter generated by the ultrasonic atomization method was inherently dependent on the ultrasonic frequency and the physical properties of the liquid [43]. In this study, neither the ultrasonic frequency nor the physical properties of the cutting fluid were influenced by variations in air pressure, thus ensuring that the droplet diameter remained essentially stable. Similarly, the standard deviation of droplet diameter followed a comparable trend of variation as the air pressure varied, as depicted in Figure 10b. Specifically, under AACF conditions, the standard deviation of droplet diameter decreased from 29.1 μm to 9 μm as the air pressure increased from 140 kPa to 300 kPa. Conversely, under UACF conditions, there was little variation in the standard deviation with increasing air pressure. Notably, UACF exhibited a lower standard deviation than AACF, indicating that the UACF can enhance the uniformity of droplet size distribution compared to the AACF process.

Additionally, the statistical results of droplet number density are presented in Figure 10c. At an air pressure of 140 kPa, the droplet number densities under AACF and UACF conditions were 5 mm^−2^ and 10 mm^−2^, respectively. When the air pressure reached 300 kPa, the droplet number densities under AACF and UACF conditions increased to 23 mm^−2^ and 26 mm^−2^, respectively. The droplet velocity was mainly responsible for this phenomenon, where high air pressure resulted in an increase in droplet velocity, thus increasing the number of droplets reaching the deposition surface per unit time. In addition, high air pressure also reduced the droplet diameter, leading to an increase in droplet number density under AACF conditions. It was also observed that the droplet number density of UACF was higher than that of AACF, which was attributed to the improved uniformity of droplet size.

**Figure 11:**
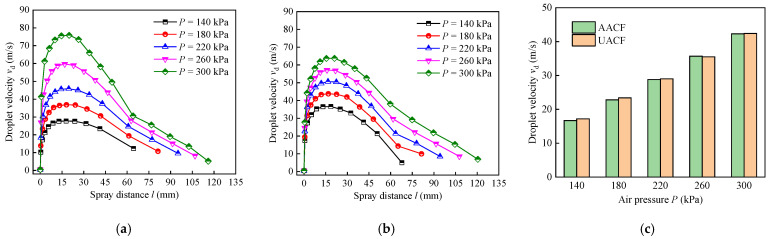
Droplet velocity under different air pressures: (**a**) variation of droplet velocity with spray distance under AACF, (**b**) variation of droplet velocity with spray distance under UACF, and (**c**) droplet velocity at a spray distance of 55 mm.

The influence of varying air pressures and spray distances on the droplet velocity under AACF and UACF is illustrated in Figure 11. Specifically, Figure 11a displays the variation in droplet velocity as a function of spray distance at five levels of air pressure under AACF conditions. At a constant air pressure, the droplet velocity rapidly attained its peak and subsequently underwent a gradual decrease with further increases in spray distance. Similarly, the droplet velocity exhibits the same pattern of variation under UACF conditions (Figure 11b). Subsequently, the droplet velocities at a spray distance of 55 mm under different air pressures for AACF and UACF were compared in Figure 11c. It was observed that the droplet velocity in AACF increased from 16.7 m/s to 42.4 m/s as the air pressure varied from 140 kPa to 300 kPa. Furthermore, there was negligible variation in droplet velocity between AACF and UACF at a fixed level of air pressure. This situation allows for a good comparison of the effect of different atomization methods (i.e., air atomization and ultrasonic atomization) on cooling and lubrication performance.

### 4.2. Effect of Air Pressure on Thickness of Liquid Film

As previously stated in reference [44], the spray characteristic parameters significantly affected the thickness of the liquid film, which provided effective cooling and lubrication for the cutting process. Therefore, the thickness of the liquid film for different combinations of spray characteristic parameters was evaluated. It was assumed that the volume loss of droplets resulting from evaporation was negligible.

For the droplet deposition region, the total droplet volume can be expressed as follows:

(15) $$V=\frac{4}{3}\pi {\left(\frac{d_{a}}{2}\right)}^{3}N_{d}s$$

where *d*_a_ represents the average droplet diameter, *N*_d_ is the droplet number density, and *s* stands for the area of droplet deposition region.

The standard deviation of droplet diameter also affected the thickness of the liquid film. That is, the larger the standard deviation of droplet diameter, the thinner the resulting liquid film becomes. Additionally, the increased droplet velocity promoted the spreading of the droplets, causing a decrease in the thickness of the liquid film. Therefore, the thickness of liquid film on the region of droplet deposition can be mathematically expressed as follows:

(16) $$h_{d}=\frac{CV}{s\sigma_{d}v_{d}}=\frac{C\pi d_{a}^{3}N_{d}}{6\sigma_{d}v_{d}}$$

where *C* is a constant, and *σ*_d_ is the standard deviation of droplet diameter.

Since *C* is an unknown constant, a dimensionless number was utilized to characterize the thickness of the liquid film. The dimensionless number *λ* was defined by Equation (17).

(17) $$\lambda =\frac{h_{d}}{h_{0}}$$

where *h*_0_ is the thickness of the liquid film at an air pressure of 140 kPa under AACF.

**Figure 12:**
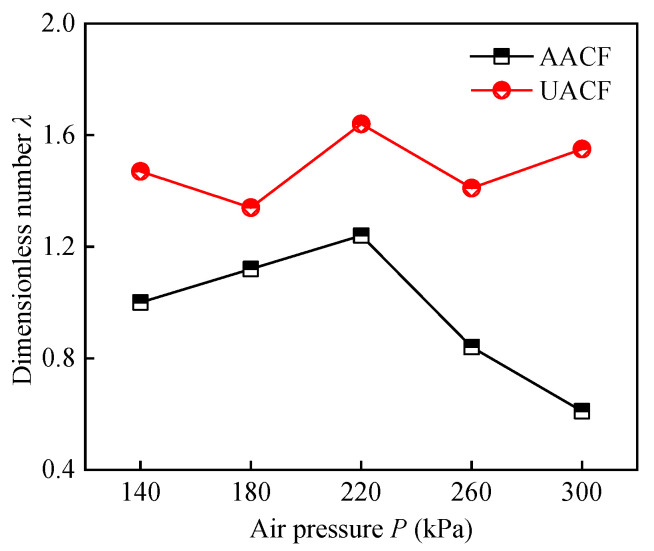
Effect of air pressure on the dimensionless number under AACF and UACF.

Figure 12 displays the effect of air pressure on the dimensionless number under AACF and UACF conditions. For the AACF condition, the dimensionless number initially showed an increasing trend, then followed by a decreasing trend. The dimensionless number in the UACF process presented a fluctuating variation trend, ranging between 1.3 and 1.7. Notably, under both AACF and UACF conditions, a higher dimensionless number can be achieved at an air pressure of 220 kPa. According to Equation (17), the thickness of the liquid film varies directly with the dimensionless number. This implied that an air pressure of 220 kPa can result in a higher thickness of the liquid film. In other words, the appropriate range of spray characteristic parameters contributed to the formation of liquid film. Furthermore, the UACF process exhibited a higher thickness of liquid film than AACF, suggesting that the ultrasonic atomization method can effectively control the spray characteristic parameters and enhance the formation of liquid film.

### 4.3. Effect of Air Pressure on Adhesion Friction Coefficient and Worn Surface

**Figure 13:**
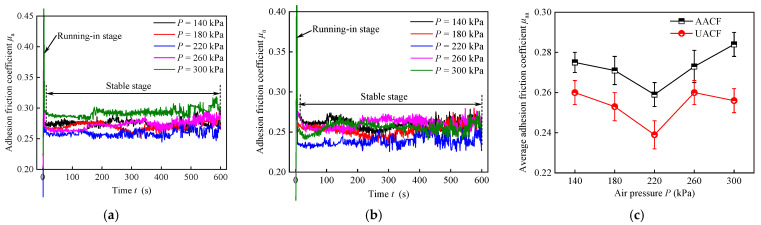
Effect of air pressure on the adhesion friction coefficient under different cooling conditions: (**a**) adhesion friction coefficient curve under AACF, (**b**) adhesion friction coefficient curve under UACF, and (**c**) variation of average adhesion friction coefficient with air pressure in the stable stage.

Figure 13 demonstrates the influence of air pressure on the adhesion friction coefficient under AACF and UACF conditions. Figure 10a,b plot the curves of adhesion friction coefficient for AACF and UACF conditions, respectively. Obviously, the adhesion friction coefficient curve can be divided into two stages: the running-in stage and the stable stage. After the initial running-in stage of the sliding process, the adhesion friction coefficient curves exhibited slight fluctuation and entered the stable stage from about 5 s. Subsequently, the average adhesion friction coefficient in the stable stage at different air pressures is summarized in Figure 10c. In the AACF condition, when the air pressure increased from 140 kPa to 220 kPa, the average adhesion friction coefficient decreased from 0.275 to 0.259. Then, it gradually rose again to reach a value of 0.284 when the air pressure reached 300 kPa. The UACF condition exhibited the same pattern of variation in average adhesion friction coefficient. Compared to AACF, UACF can reduce the adhesion friction coefficient by 4.7% to 9.8%. According to the results in Figure 12, it can be found that the higher the dimensionless number, the lower the average adhesion friction coefficient. This result indicated that an increase in the thickness of liquid film contributed to reducing the adhesion friction coefficient.

**Figure 14:**
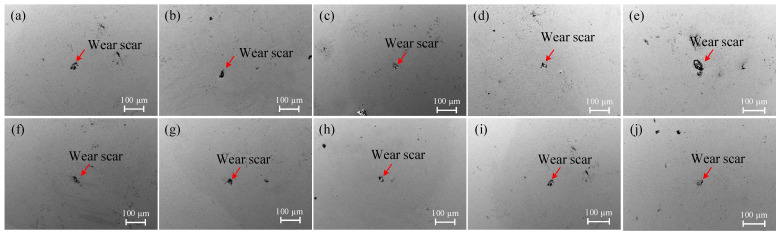
Effect of air pressure on the worn surface of the WC ball under (**a**–**e**) 140 kPa, 180 kPa, 220 kPa, 260 kPa, and 300 kPa for AACF, (**f**–**j**) 140 kPa, 180 kPa, 220 kPa, 260 kPa, and 300 kPa for UACF.

Additionally, the worn surfaces of WC balls were detected, as shown in Figure 14. It was noted that the surfaces of the WC balls exhibited small wear scars under AACF and UACF conditions, regardless of the air pressure level. However, there was no significant difference in the wear scars. This phenomenon was attributed to the fact that the 10-min duration was not enough to cause serious wear on the surface of the WC ball. In the future, discontinuous sliding tests will be conducted with a longer duration to further study the wear process of the WC balls.

## 5. Conclusions

In this work, the spray characteristic parameters under AACF and UACF with different air pressures were evaluated in terms of droplet size distribution, droplet velocity, and droplet number density. Furthermore, the impact of spray characteristic parameters on the thickness of the liquid film and adhesion friction coefficient was investigated. The following conclusions have been drawn:

(1) The average value and standard deviation of droplet diameters were used to describe the size distribution of droplets. Under AACF conditions, an increase in air pressure reduced the average droplet diameter by 52.6% and the standard deviation of droplet diameter by 69%, respectively. However, under the UACF process, only a reduction of 6% and 17.6% was found in the average droplet diameter and standard deviation of droplet diameter, respectively. The air pressure in the UACF process has minimal effect on the size distribution of droplets compared to AACF condition.

(2) When the air pressure increased from 140 kPa to 300 kPa, the droplet number density in the AACF condition increased from 5 mm^−2^ to 23 mm^−2^, and the droplet number density in the UACF condition increased from 10 mm^−2^ to 26 mm^−2^. UACF exhibited a higher droplet number density than AACF due to the improved uniformity of droplet size distribution produced by the ultrasonic atomization method. Additionally, with the increase in air pressure, the droplet velocity exhibited an increasing trend, regardless of cooling conditions.

(3) Both AACF and UACF conditions yielded a higher thickness of liquid film at an air pressure of 220 kPa. The average droplet diameter, standard deviation of droplet diameter, droplet number density, and droplet velocity are the important parameters affecting the thickness of the liquid film.

(4) Compared to AACF, UACF could reduce the adhesion friction coefficient by 4.7–9.8%. The appropriate combination of spray characteristic parameters in UACF facilitates the formation of liquid film, thereby reducing the adhesion friction coefficient and improving friction conditions.

## Figures and Tables

**Table 1:** Cooling parameters under AACF and UACF.

Contents	Value
Air pressure *P* (kPa)	140, 180, 220, 260, 300
Flow rate of cutting fluid *Q* (mL/min)	15
Concentration of cutting fluid *W* (%)	12

**Table 2:** Mechanical properties of the 15Cr14Co12Mo5Ni2 ultra-high-strength steel.

Contents	Value
Tensile strength *σ*_b_ (MPa)	1780
Yield strength *σ*_s_ (MPa)	1380
Density *ρ* (kg/m^3^)	7960
Hardness (HRC)	35
Fracture toughness *K*_IC_ (MPa·^1/2^)	75

## Data Availability

Dataset available on request from the authors.
